# CAREGIVER CONTRIBUTION TO OCCUPATIONAL THERAPY VIDEO VISITS

**DOI:** 10.1093/geroni/igac059.020

**Published:** 2022-12-20

**Authors:** Megan Gately, Dylan Waller, Emily Metcalf, Lauren Moo

**Affiliations:** VA Bedford Health Care System, Bedford, Massachusetts, United States; VA Portland Health Care System, Portland, Oregon, United States; VA Bedford Health Care System, Bedford, Massachusetts, United States; VA Bedford Health Care System, Bedford, Massachusetts, United States

## Abstract

Caregivers’ role facilitating older adults’ participation in diverse health care services delivered using video telehealth (i.e., live sessions) is not well-understood. This study surveyed occupational therapy (OT) practitioners across Veterans Health Administration (VHA) about caregiver participation in VA Video Connect (VVC), VHA’s videoconferencing platform. 293 OT practitioners participated in the survey, with 47% reporting that caregivers participated in VVC often. The foremost reported patient factors necessitating caregiver participation in video visits were patient lack of technical skills (76%) and cognitive impairment (72%). Barriers to caregiver participation in video visits included poor connectivity and caregivers’ own age or health related impairments, while benefits included increased collaboration with family (87%). This study enhances our understanding of caregivers’ participation in video telehealth, highlighting factors driving caregiver participation and suggesting strategies to optimize this service delivery format for older adults.

